# Amplification of Replication Competent HIV-1 by Adoptive Transfer of Human Cells From Infected Humanized Mice

**DOI:** 10.3389/fcimb.2020.00038

**Published:** 2020-02-11

**Authors:** Hang Su, Sruthi Sravanam, Santhi Gorantla, Rafal Kaminski, Kamel Khalili, Larisa Poluektova, Howard E. Gendelman, Prasanta K. Dash

**Affiliations:** ^1^Department of Pharmacology and Experimental Neuroscience, College of Medicine, University of Nebraska Medical Center, Omaha, NE, United States; ^2^Department of Neuroscience, Lewis Katz School of Medicine at Temple University, Philadelphia, PA, United States; ^3^Department of Pharmaceutical Sciences, Center for Neurovirology, College of Pharmacy, University of Nebraska Medical Center, Omaha, NE, United States

**Keywords:** HIV-1 infection, viral latency, humanized mice, viral outgrowth assays, adoptive transfers, viral reservoirs, lymphoid tissue

## Abstract

Detection of latent human immunodeficiency virus type 1 (HIV-1) in “putative” infectious reservoirs is required for determining treatment efficiency and for viral elimination strategies. Such tests require induction of replication competent provirus and quantitative testing of viral load for validation. Recently, humanized mice were employed in the development of such tests by employing a murine viral outgrowth assay (mVOA). Here blood cells were recovered from virus infected antiretroviral therapy suppressed patients. These cells were adoptively transferred to uninfected humanized mice where replication competent virus was recovered. Prior reports supported the notion that an mVOA assay provides greater sensitivity than cell culture-based quantitative VOA tests for detection of latent virus. In the current study, the mVOA assays was adapted using donor human hematopoietic stem cells-reconstituted mice to affirm research into HIV-1 elimination. We simulated an antiretroviral therapy (ART)-treated virus-infected human by maintaining the infected humanized mice under suppressive treatment. This was operative prior to human cell adoptive transfers. Replication-competent HIV-1 was easily detected in recipient animals from donors with undetectable virus in plasma. Moreover, when the assay was used to investigate viral presence in tissue reservoirs, quantitative endpoints were determined in “putative” viral reservoirs not possible in human sample analyses. We conclude that adoptive transfer of cells between humanized mice is a sensitive and specific assay system for detection of replication competent latent HIV-1.

## Introduction

Antiretroviral therapy (ART) has transformed human immunodeficiency virus type one (HIV-1) infection from a life-threatening disease into a life-long chronic condition. Though ART maintains viral suppression and preserves immune function it demands strict regimen adherence with associated stigmas, costs, resistance and inherent drug toxicities. Therefore, complete HIV-1 sterilization has been sought in order to end the epidemic (Deeks et al., 2016). Nonetheless, after almost 40 years since the discovery of HIV-1, viral cure has been reported in two cases only (Hutter et al., 2009; Gupta et al., 2019). The major barrier for “cure” rests in the ability to eliminate HIV-1 latency. This will require the clearance of few numbers of infected cells present during the disease course following treatment where cell populations emerge that contain replication-competent latent HIV-1 DNA. These can elicit progeny virus production that follows cell activation (Eriksson et al., 2013; Darcis et al., 2019). Thus, while research into viral elimination strategies show promise to eliminate the latent viral reservoir, highly sensitive and specific validations are required for HIV-1 detection. Such tests enable measurements of the size, location and distribution of any residual virus in tissues, in cells and in body fluids (Laird et al., 2013; Rouzioux and Richman, 2013; Gupta et al., 2017; Sanyal et al., 2017). Evidence for “cure” is certainly required before enacting treatment interruption (Persaud et al., 2013; Henrich et al., 2014). Current assays for determining HIV-1 reservoirs have focused on viral amplification based on polymerase chain reaction (PCR) tests for detection of viral-specific sequences (Bruner et al., 2015; Massanella and Richman, 2016). While PCR analysis can detect as low as 1 viral copy (Tosiano et al., 2019) they fail to distinguish intact and defective virus. As the majority of viral genomes are defective such tests would largely overestimate HIV-1 reservoir size (Eriksson et al., 2013; Bruner et al., 2016). Therefore, full genome assays are required to distinguish defective from intact proviruses. These have been incorporated in the measurement of true HIV-1 reservoirs (Bruner et al., 2019). Alternatively, quantitative viral outgrowth assays (qVOA) are required as they detect only replication-competent virus. Such assays remain as the gold standard for measures of replication competent latent HIV-1 (Siliciano and Siliciano, 2005; Baxter et al., 2016; Grau-Exposito et al., 2017). A major challenge to the qVOA assay is that it can only reactivate a small portion of HIV-1 reservoirs, as larger scale of virus detection would require repetitive stimulations (Ho et al., 2013; Hosmane et al., 2017). Therefore, qVOA would underestimate the complete HIV-1 reservoir size (Persaud et al., 2013; Henrich et al., 2014). This has led to the development of alternative viral detection systems (Metcalf Pate and Blankson, 2017; Schmitt and Akkina, 2018).

As HIV-1 exclusively infects humans and chimpanzees, humanized mice are alternatives to reflect virus-human cell interactions (Araínga et al., 2016; Victor Garcia, 2016; Marsden and Zack, 2017; Su et al., 2019). Recent studies demonstrated that humanized mice can be used to recover HIV-1 from ART-suppressed donor cells (Metcalf Pate et al., 2015; Charlins et al., 2017; Henrich et al., 2017; Yuan et al., 2017; Salgado et al., 2018). In these assay systems HIV-1 was successfully isolated from recipient mice from infected donors with undetectable plasma viral loads. The murine-based VOA (mVOA) enables replication competent HIV-1 to be recovered from parallel samples that were previously tested negative by qVOA. This suggested that mVOA is a more sensitive assay for viral detection (Charlins et al., 2017). Based on these reports, we adapted the mVOA assay using donor humanized mice (Dash et al., 2019). In this study we validated an HIV “cure” strategy using infected humanized mice treated sequentially with long-acting slow-effective release ART (LASER ART) and CRISPR-Cas9 followed with 9 weeks of treatment withdrawal. LASER ART was first administered to maximize HIV-1 restriction and reduce the proviral DNA load followed with CRISPR-Cas9 used to target conserved LTR and Gag regions to excise remaining proviral DNA (Hu et al., 2014; Gendelman et al., 2019). At study end point 3/6 randomly selected humanized mice that received LASER ART and CRISPR-Cas9 treatment were virus suppressed while animals with no or single treatment showed detectable virus and viral rebound. To confirm HIV-1 elimination from the three animals (M3319, M3324, and M3336, Table S1) splenocytes and bone marrow (BM) cells were collected and separately engrafted into naïve humanized mice. Recipients adoptively transferred from M3319 and M3336 mice showed no recovery of progeny HIV-1. However, replication-competent virus was detected in control untreated, single treated or dual treated animals (including M3324) where viral rebound was demonstrated. In these mice HIV-1 persisted in cell-tissue reservoirs. The results were recorded even when plasma viral load (VL) remained undetectable. In a parallel study, we simulated clinical ART treatment by maintaining infected mice on LASER ART until plasma VLs reached levels at or below the detection limits. In these tests peripheral blood mononuclear cells (PBMCs) and splenocytes were collected from infected and treated animals then adoptively transferred into naïve humanized mice. The results showed that virus can be readily recovered demonstrating that adoptive transfer between humanized mice is an effective means to recover residual HIV-1 that includes suppressive ART treatments.

## Materials and Methods

### Generation, HIV-1 Infection and Treatment of Humanized Mice

NSG mice were purchased from the Jackson Laboratory (Bar Harbor, ME) and bred under pathogen-free conditions in compliance with ethical guidelines required by the National Institutes of Health and the University of Nebraska Medical Center. All experimental protocols were approved by the University of Nebraska Medical Center Institutional Animal Care and Use Committee. All animal studies were performed according to UNMC institutional policies and the National Institutes of Health guidelines.

**Figure 1:**
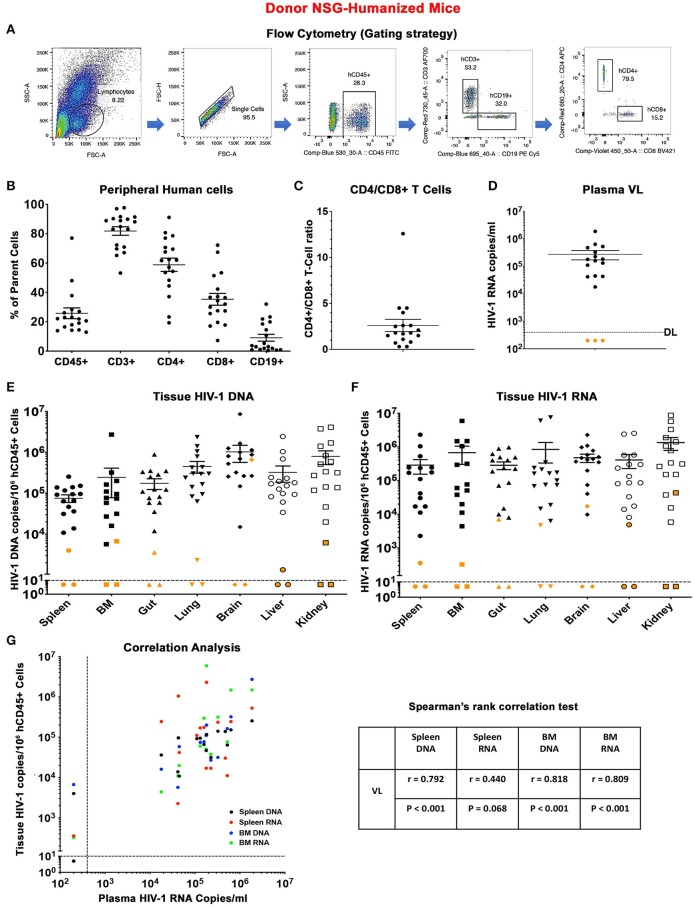
Descriptors of donor humanized mice. **(A)** Flow cytometric gating strategy for human cell reconstitution in NSG-humanized mice. In brief, human cells (hCD45+) were gated from total lymphocytes and single cells and subsequently grouped into human T lymphocytes (hCD3+) and B lymphocytes (hCD19+). Human CD3+ cells were separated into human CD4+ (hCD4+) and CD8+ (hCD8+) T lymphocytes. Detailed immune and viral information of individual animal (*n* = 18) was presented as scatter dots covering **(B)** peripheral human CD45+, CD3+, CD4+, CD8+, and CD19+ cell levels, **(C)** CD4+/CD8+ T cell ratio, **(D)** plasma viral load (pVL), **(E)** tissue HIV-1 DNA, and **(F)** tissue HIV-1 RNA distributed across spleen, BM, gut, lung, brain, liver, and kidney. Data are expressed as mean ± SEM. Orange dots in **(D)** indicated that pVL was below the detection limit (DL) of 400 HIV-1 RNA copies/ml as shown by the dotted line. Orange dots in **(E,F)** shows tissue viral levels of animals with undetected pVL. The DL is below 10 HIV-1 copies/10^6^ human CD45+ cells as measured using semi-nested real time qPCR. The dotted line corresponded to the DL. **(G)** Spearman's rank correlation tests were conducted between humanized mice pVL and respective spleen DNA (black), spleen RNA (red), BM DNA (blue), and BM RNA (green). The Spearman correlation coefficient (*r*) and *p*-value for each analysis were displayed.

Human CD34+ hematopoietic stem cells (HSCs) were enriched from umbilical cord blood using immunomagnetic beads according to manufacturer instructions (CD34+ selection kit, Miltenyi Biotec Inc., Auburn, CA). Hu-HSC mice were generated by intrahepatic injection of human CD34+ HSCs into new-born NSG pups that received 1Gy of irradiation (RS 2000 X-ray Irradiator, Rad Source Technologies, Buford, GA,) (Gorantla et al., 2010, 2012b). Human cell reconstitution was monitored monthly for 5–6 months by flow cytometry (Figure 1A) (Dash et al., 2012). At 20–24 weeks of age, hu-HSC mice following affirmation of human cell reconstitution were intraperitoneally infected with HIV-1_NL4−3_ (Adachi et al., 1986) or HIV-1_ADA_ (Gendelman et al., 1992). Stocks of HIV-1_NL4−3_ and HIV-1_ADA_ were made by propagation with CD4+ T cells and monocyte-derived macrophages (MDMs), respectively. Both viral titers were 10^4^ tissue culture infection dose_50_ (TCID_50_)/animal (see Table S1). Viral infection was confirmed at week 2 by peripheral HIV-1 RNA assays [automated COBAS Ampliprep System V2.0/Taqman-48 system, (with a detection limit of 20 copies/ml) Roche Molecular Diagnostics, Basel, Switzerland] according to the manufacturer's instructions. For this assay, 50–100 μl of mouse serum was diluted to 1 ml sterile filtered normal human serum. The detection limit of the assay after dilution is <400 viral RNA copies/ml, based on the initial plasma used for testing. In study where hu-HSC mice (including M3319, M3324, and M3336) were adopted, in part, from a prior report (Dash et al., 2019) and animals were randomly distributed into three groups. Group 1, HIV-1 infected control; Group 2, HIV-1 infected and LASER ART [long-acting (LA) dolutegravir (DTG), rilpivirine (RPV), lamivudine (3TC), and abacavir (ABC)] treated (Singh et al., 2016; Guo et al., 2017; Sillman et al., 2018; Dash et al., 2019); Group 3, HIV-1, LASER ART and AAV_9_-CRISPR-Cas9 treatments (Kaminski et al., 2016). The LA nanoformulated DTG and RPV were injected monthly at doses equivalent to 45 mg/kg of parent drugs and ABC and 3TC were administered weekly for 2 months at doses equivalent to 50 mg/kg of parent drugs. A single dose of AAV_9_-CRISPR-Cas9 was injected intravenously at 10^12^ GC (engineering equivalent) units, 1 week after last dose of LASER ART administration. Eight weeks later (total 9 weeks) after LASER ART interruption, donor hu-HSC mice were sacrificed and analyzed for immune cell profiles and viral infection. Splenocytes and bone marrow (BM) cells were isolated from donor animals and assessed for cell viability by trypan blue and live/dead stains. Cell counts were recorded using the TC-20 automated cell counter (Bio-Rad, Hercules, CA). The whole cell population containing human cells from donor splenocytes and BM were separately injected into respective naïve humanized mice intraperitoneally immediately after cells were counted. In the second study, all three donor humanized mice were infected with HIV-1_ADA_ at 10^4^ TCID_50_ for 2 weeks before LASER ART [nanoformulated myrisolyated cabotegravir CAB, 3TC and ABC (NMCAB, NM3TC, and NMABC and nanoformulated RPV (NRPV) (Zhou et al., 2018)] administration. NMCAB and NRPV were injected monthly at doses equivalent to 45 mg/kg of parent drugs and NMABC and NM3TC were injected weekly at doses equivalent to 50 mg/kg of parent drugs. LASER ART was maintained for 3 months before animal sacrifice. Donor PBMCs and splenocytes were isolated, counted, analyzed by flow cytometry for the quantification of human cells, and separately engrafted into naïve humanized mice to assess HIV-1 recovery.

### Adoptive Transfer

Naïve recipient hu-HSC mice with sustained levels of human cell reconstitution were selected for allograft as previously reported (Gorantla et al., 2007, 2012a; Su et al., 2019). To preclude loss of human cell numbers in ART suppressed animals, total cell population were isolated and transferred into recipient animals. In the first study, recipients were maintained for 4 weeks after engraftment while in the second study the recipients were maintained for 9 weeks before animal sacrifice.

### Flow Cytometry

Peripheral blood was collected from facial vein for routine monitoring or cardiac puncture at animal sacrifice. Blood was collected in ethylenediaminetetraacetic acid (EDTA)-coated tubes and incubated with pan-human monoclonal antibodies to CD45, CD3, CD19, CD4, and CD8 (BD Pharmingen, San Diego, CA). Flow cytometry was operated on BD LSRII (BD Immunocytometry Systems, Mountain View, CA) system and data were analyzed using FlowJo software (BD Pharmingen, San Diego, CA).

### Tissue Nucleic Acid Extraction and Viral Quantification

Animal tissues (brain, lung, liver, spleen, BM, gut, and kidney) were collected and homogenized by Qiagen Tissue Lyser II (QIAGEN, Hilden, Germany). Total cellular DNA and RNA were extracted using Qiagen All Prep DNA/RNA Mini Kit (QIAGEN, Hilden, Germany). Tissue HIV-1 RNA was reverse transcribed to complementary DNA using Thermo-Fisher Scientific Verso cDNA Synthesis Kit (Invitrogen, MA). HIV-1 DNA and RNA were quantified by semi-nested real-time PCR as previously described (Araínga et al., 2016). In brief, the first round of PCR was performed on a conventional PCR machine (T100 Thermal Cycler, Bio-Rad, CA) in 25 μl of PCR reaction mix containing 500 ng of template and 50 ng each of both primers annealing to HIV-1 gag region and the reaction conditions are as follows: 94°C for 3 min, followed by 15 cycles of 94°C for 30 s, 55°C for 30 s, and 72°C for 1 min. The product of the first PCR was subsequently used as a template in the second semi-nested real-time PCR amplification. The assay was performed on the ABI Step One Plus real-time PCR machine (Applied Biosystems, Foster City, CA) using TaqMan detection probe and primers. Five microliter of the first PCR product was diluted to 10 μl with PCR master mix containing two primers at 0.2 μM each and 0.2 μM TaqMan dual-labeled fluorescent probe. Real-time PCR settings were as follows: 50°C for 2 min, then 95°C for 10 min, followed by 40 cycles of 95°C for 15 s, and 60°C for 1 min. DNA isolated from ACH-2 cells containing one integrated copy of HIV-1 per cell was used as standard in serial 10-fold dilutions with HIV-1 copy numbers ranging from 10^1^ to 10^5^ DNA copies/reaction. The detection limit for this analysis is below 10 HIV-1 DNA copies/10^6^ human CD45+ cells. Human CD45 (Hs0036534_g1) (Life Technology, California, USA) was applied as reference gene to quantify human cell numbers in tested samples. Final HIV-1 levels were normalized to 10^6^ human CD45+ cells.

### Immunohistochemistry

Tissue samples were collected and fixed with 4% paraformaldehyde for 24 h then processed followed by paraffin embedding. Five-micron thick tissue sections were collected and immuno-stained with mouse monoclonal antibodies against human HLA-DQ/DP/DR (clone CR3/43, 1:100, DAKO, Carpinteria, CA) and HIV-1 p24 (1:10, DAKO, Carpinteria, CA). The polymer-based horse radish peroxidase (HRP) and 3,3′-Diaminobenzidine (DAB) DAKO EnVision system was used for staining development. The nuclei were counterstained with Mayer's hematoxylin (Dash et al., 2012). Images were acquired with a Nuance EX camera fixed to a Nikon Eclipse E800 microscope using Nuance software (Cambridge Research & Instrumentation, Woburn, MA). Human HLA-DR images were obtained at 20 x magnifications and HIV-1p24 images were captured at 40 x objective magnifications.

### Statistical Analyses

Data were analyzed using GraphPad Prism 8.0 software (La Jolla, CA) and results were presented as the means ± the standard error of the mean (SEM). The Student's *t*-test was used for two-group comparison (two-tailed) while a value of *p* < 0.05 was considered statistically significantly different. Association between two parameters was conducted using two-tailed Spearman's rank correlations.

## Results

### Donor hu-HSC Mice

In an initial experiment, donor humanized mice that received either single or dual treatment were studied for 9 weeks off treatment to assay evidence for HIV-1 infection. Human cells from tissues were harvested for adoptive transfer (Figure S1). A total of 18 donor humanized mice were included. These were 11 males and seven females (Table S1). The median age at allograft was 37 (34–45) weeks. Human cells were readily detected in the donor animals throughout the study with a mean level of human CD45+ cells at 25.8% ± 3.7, CD3+ cells at 81.8% ± 2.9, CD4+ cells at 58.8% ± 4.5, CD8+ cells at 35.3% ± 4.0, and CD19+ cells at 9.1% ± 2.4 (Figures 1A,B). The mean CD4/CD8 T cell ratios was 2.6 ± 0.7 (Figure 1C). No significant differences were observed between male and female donors based on the level of human cell reconstitution and CD4/CD8 T cell ratios (Figures S2A,B). All HIV-1 infected, HIV-1 infected and LASER ART, and “select” dual treated animals showed viral rebound after treatment interruption (Table S1). The mean plasma VL of donor humanized mice was 2.7 ± 1.0 × 10^5^ HIV-1 RNA copies/ml (Figure 1D). Notably, three dual-treated animals (M3319, M3324, and M3336) were virally suppressed (Table S1 and Figure 1D). Sex difference did not affect plasma viral levels (Figure S2C). A range of tissues (spleen, BM, gut, lung, brain, liver, and kidney) were harvested to evaluate evidence for residual HIV-1. Tissue HIV-1 compartments were established in animals that showed plasma VL (Figures 1E,F). Interestingly, while tissue HIV-1 remained below the limits of detection in M3319 and M3336, viral DNA and RNA was observed in M3324, demonstrating that HIV-1 persisted in tissues even when peripheral viral amplification was not observed (Figures 1E,F). Tissue viral levels between male and female donors were not changed (Figures S2D,E). Correlations between donor plasma VL and spleen and BM viral burdens were observed (Figure 1G). Overall, peripheral HIV-1 levels proved to be a viable indicator of tissue viral production. However, residual virus could be present in tissues when peripheral VL was absent and precluding HIV-1 elimination.

### Recipient hu-HSC Mice

**Figure 2:**
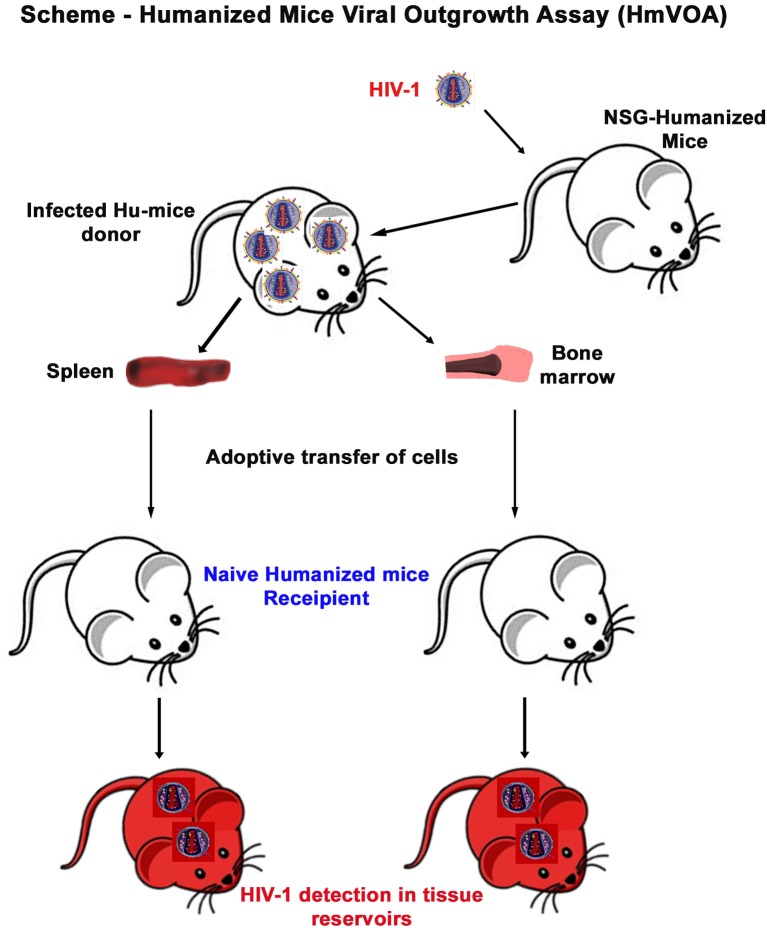
Murine HIV-1 outgrowth assay. A mixture of mouse and human cells were isolated from donor humanized mouse spleen or BM and immediately intraperitoneally injected into naïve humanized mice as described in the scheme. Recipient animals were maintained for 1 month before sacrifice and evaluated for HIV-1 recovery.

**Figure 3:**
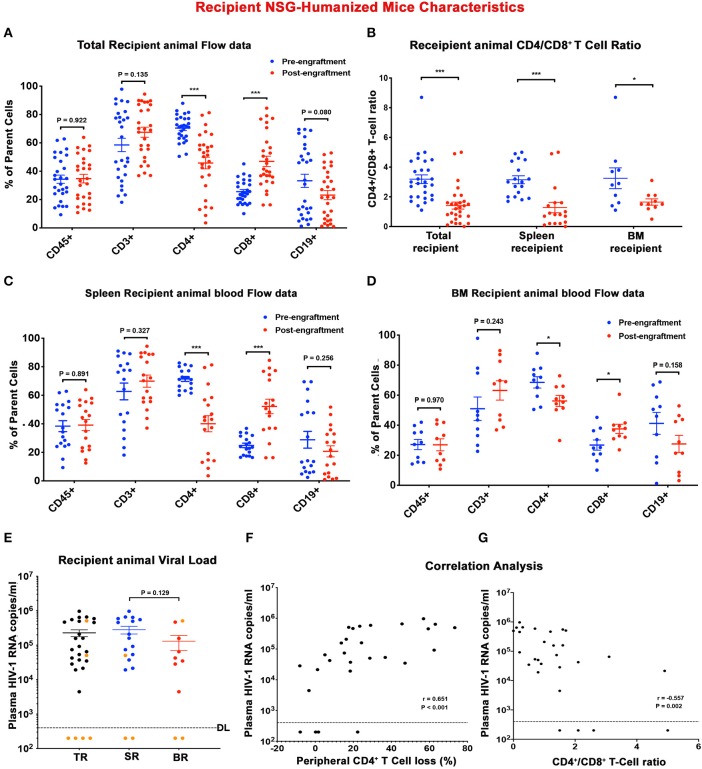
Descriptors of recipient humanized mice. Peripheral human cell levels (hCD45+, hCD3+, hCD4+, hCD8+, and hCD19+ cells) before (blue) and after (red) adoptive transfer were depicted for **(A)** total recipient (TR) humanized mice, and subgroups **(C)** spleen recipient (SR) and **(D)** bone marrow recipient (BR) humanized mice. **(B)** Scatter plots represented CD4+/CD8+ T cell ratio of individual TR, SR, and BR before (blue) and after (red) adoptive transfer. **(E)** Plasma viral load (pVL) of individual recipient humanized mouse at sacrifice was displayed for TR (black), SR (blue), and BR (red). Orange dots represented humanized mice receiving engraftment from donors with undetected plasma VL. The detection limit is <400 HIV-1 RNA copies/ml. A Spearman's rank correlation test was conducted to investigate the association **(F)** between recipient humanized mouse plasma VL and respective peripheral CD4+ T cell loss and **(G)** between recipient humanized mouse plasma VL and respective end-point peripheral CD4+/CD8+ T cell ratio. The Spearman correlation coefficient (*r*) and *p*-value for each analysis were displayed. In **(A–E)**, data are expressed as mean ± SEM and considered *, *** statistically significant, at *p* < 0.05 and *p* < 0.001.

To determine the presence of replication-competent cell-based tissue HIV-1 in donor animals, single cell suspensions were isolated from spleen and BM viral reservoir sites. Isolated cells were then adoptively transferred to naïve reconstituted humanized mice (recipients) to assess whether viral recovery was possible (Figure 2). A total of 28 naïve humanized-NSG mice were employed as recipients in the current report. Among them 18 animals received splenocytes engraftment and 10 animals were adoptively transferred with BM cells (Figure 2 and Table S2). The median age of the recipient animal at allograft was 30 (25 ~ 37) weeks. Pre-engraftment humanization of individual recipient animals was shown in Figures 3A–D (blue dots) and Table S3. According to sample availability, donor splenocytes and BM cells were independently injected into the recipient animals by intraperitoneal injection. The engraftment contained a mixture of human- and mouse-originated cells. The median of viable engrafted cell count was 9.6 (5.1–19.8) million for spleen and 8.7 (4.3–10.7) million for bone marrow (Table S2). Engraftment was well tolerated by all recipient animals. Four weeks after adoptive transfer, recipient humanized mice were sacrificed and evaluated for HIV-1 recovery. Peripheral human CD45+, CD3+ T cells, and CD19+ B cells were not significantly changed in TR, SR, and BR between pre- and post-engraftment. Peripheral CD4+ T cells were significantly decreased in post- compared to pre-engraftment levels and CD8+ T cells were increased (Figures 3A,C,D and Table S3). CD4/CD8+ T cell ratios, as a major indicator of immune function and long-term clinical outcomes (Lu et al., 2015), declined in all recipients (Figure 3B and Table S3). Recipient humanized mice amplified HIV-1 in tissues from donors including M3324, demonstrating that the residual viruses in these animals were replication-competent. However, in the animals transplanted from “virus-free” donors (M3319 and M3336), HIV-1 was not detected (Figure 3E and Table S2). Plasma VL showed no significant differences between spleen and bone marrow cells recipients (Figure 3E and Table S3). Peripheral viral replication was positively correlated with CD4+ T cell loss in TR (calculated as pre- minus post-engraftment CD4+ T cell levels) (Figure 3F). There was a negative association between plasma VL and end point CD4/CD8+ T cell ratios (Figure 3G). We observed a positive correlation of plasma VL between donor and SR but not between donor and BR (Figure S3). Tissue HIV-1 infection as measured to look for viral DNA (Figures S4A,C) and RNA (Figures S4B,D) was readily established and disseminated amongst spleens, BM, guts, lungs, brains, livers, and kidneys in allografted humanized mice except those four animals obtaining engraftment from donors M3319 and M3336 (Figures S4A–D). There were not significant differences on tissue viral burdens (except gut HIV-1 DNA) between SR and BR (Figures S4A–D). For the SR (M3404) and BR (M3406) of M3324, spleen and lymph node samples were stained for human HLA-DR and HIV-1p24. HIV-1 infection was detected in both animals (Figure S4E, shown as brown dots). Taken together, replication-competent HIV-1 persisted in tissue even when plasma VL was undetectable and that these were recaptured by mVOA.

### Recovery of HIV-1 From ART-Treated Mice

**Figure 4:**
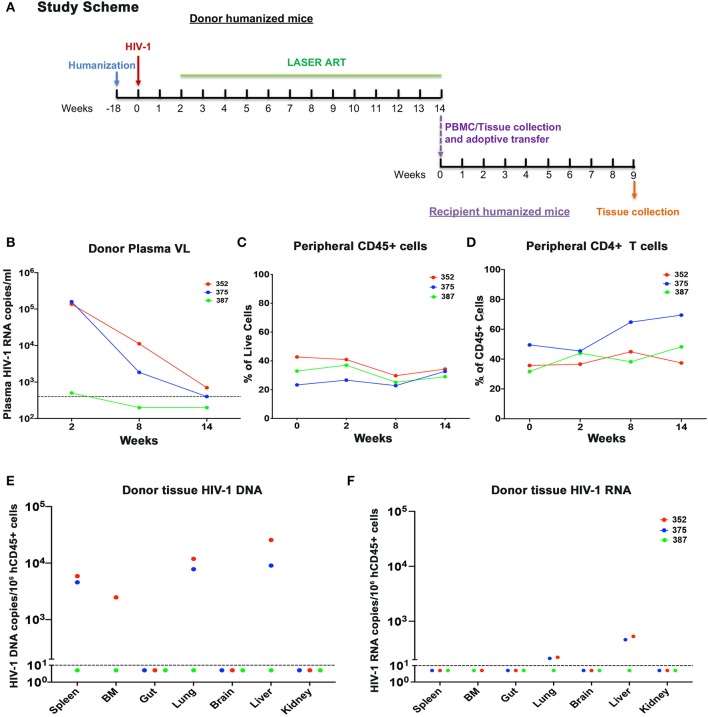
Adoptive transfer of donor humanized mice with undetected or close-to-detection limit viral load. **(A)** The experimental scheme. In brief, donor humanized mice were first infected with HIV-1_ADA_ for 2 weeks. Animals were then maintained under LASER ART which contained a combination of nanoformulated long-acting named NMCAB, NMABC, NM3TC, and NRPV for 3 months until pVL reached undetectable level or close to detection limit of 400 HIV-1 RNA copies/ml. Donor humanized mouse splenocytes and PBMCs were extracted and separately engrafted to respective naïve humanized mice. The recipient animals were maintained for 9 weeks to monitor HIV-1 recovery. The dynamic changes of individual donor humanized mouse were recorded for **(B)** pVL, **(C)** peripheral human CD45+, and **(D)** peripheral human CD4+ T cells. **(E)** Tissue HIV-1 DNA and **(F)** tissue HIV-1 RNA in individual donor humanized mice was evaluated across spleen, BM, gut, lung, brain, liver, and kidney, using semi-nested real time qPCR. The dotted line in **(B)** indicated the detection limit (DL) of 400 HIV-1 RNA copies/ml. The dotted line in **(E,F)** showed the DL below 10 HIV-1 copies/10^6^ human CD45+ cells.

**Figure 5:**
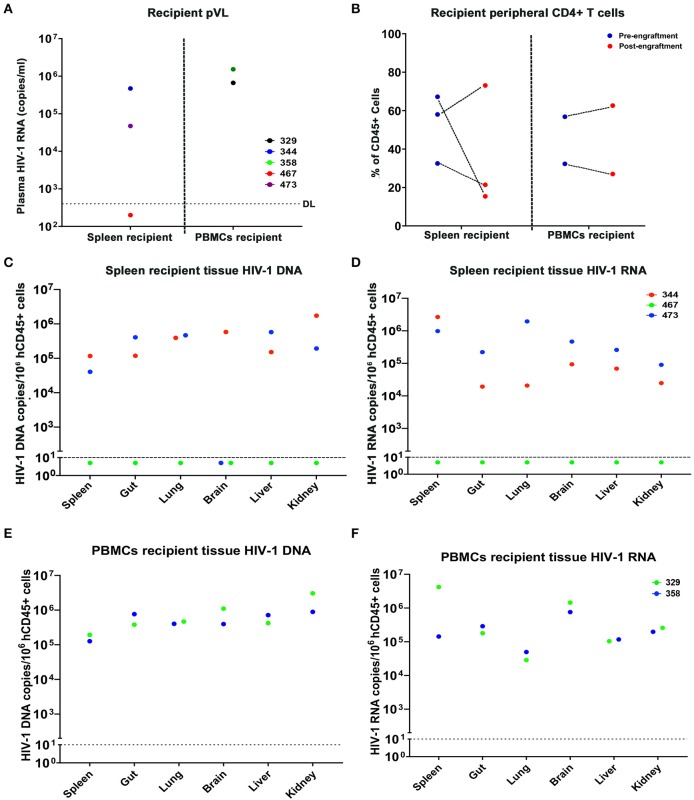
Recipient humanized mice engrafted from donor humanized mice under effective treatment. A total of 5 naïve humanized mice received adoptive transfer among which three (M344, M467, and M473) received splenocytes engraftment, and two (M329 and M358) received PBMCs engraftment. **(A)** Peripheral VL of individual spleen and PBMCs recipients after adoptive transfer, respectively. **(B)** Peripheral CD4+ T cell levels of individual spleen and PBMCs recipients after adoptive transfer. **(C,E)** tissue HIV-1 DNA and **(D,F)** tissue HIV-1 RNA in individual donor humanized mice was evaluated across spleen, gut, lung, brain, liver, and kidney, using semi-nested real time qPCR. M344 (red) received spleen transfer from donor M352. M473 and M358 (blue) received spleen and PBMCs engraftment from M375, respectively. M467 and M329 (green) received spleen and PBMCs engraftment from M387, respectively. The dotted line in **(A)** indicates the detection limit (DL) of 400 HIV-1 RNA copies/ml. The dotted line in **(C–F)** showed the DL below 10 HIV-1 copies/10^6^ human CD45+ cells.

In the first study, cells from donor virus-infected with and without treatment mice were tested 9 weeks after treatment interruption prior to adoptive transfer. Although M3324 demonstrated undetectable plasma VL at allograft HIV-1 was recovered from recipient humanized mice. To determine whether infected cells can be recovered from ART treated humanized mice a second follow-on study was conducted (Figure 4A). Three donor humanized mice (M352, M375, and M387) were infected with HIV-1_ADA_ at 10^4^ TCID_50_ for 2 weeks when plasma VL was 1.36 × 10^5^, 1.59 × 10^5^, and 500 HIV-1 RNA copies/ml, respectively (Figure 4B). LASER ART was maintained for 3 months with plasma VL at or below the detection limit (Figure 4B and Table S4). Animal body weight was consistent throughout the study for all animals (Figure S5A). No change in peripheral human CD45+ cells was recorded during the study (Figure 4C). Increases in peripheral CD4+ T cells were observed in animals M375 and M387 but not in M352 mice (Figure 4D). To demonstrate latent HIV-1 infection in both blood and tissue, PBMCs and splenocytes were isolated from the mice then engrafted into naïve humanized mice. Viable human cells were quantified using flow cytometry (Table S4). Three recipients received splenocytes engraftment and two received PBMCs engraftment from respective donors. The engraftment contained whole cell population, but an equivalent human CD45+ and CD4+ T cell counts were calculated by flow cytometry tests, before being injected for adoptive transfer (Table S5). Low levels of HIV-1 RNA were detected in lung and liver of M352 and M375 and no tissue HIV-1 RNA was observed in M387 (Figure 4F), demonstrating that active tissue viral replication was suppressed. However, persistent HIV-1 DNA was detected in spleen, BM, lung, and liver of M352 and M375 (Figure 4E), demonstrating that ART alone does not eradicate HIV-1 infection. No tissue HIV-1 DNA was observed in M387 likely due to the assay detection limit. HIV-1 was recovered in spleen recipient M344 and M473 engrafted from M352 and M375, respectively, but failed to recover in M467 engrafted from M387 (Figure 5A). In PBMCs recipient M329 and M358 engrafted from M387 and M375, respectively, HIV-1 was successfully rescued (Figure 5A). Noteworthy, no HIV-1 DNA or RNA was detected in M375 PBMCs by qPCR analysis (data not shown, M387 PBMCs were not available for study). These results demonstrated that humanized mice-based mVOA can recover HIV-1 from virally suppressed humanized mouse donors. The recipient humanized mice showed steady body weight (Figure S5B). Peripheral CD4+ T cells were consistent in recipients M344 and M473, but increased in M467 (Figure 5B). In PBMCs recipients M329 and M358, peripheral CD4+ T cells were elevated at 5 weeks and declined at 9 weeks after adoptive transfer (Figure 5B). Tissue HIV-1 DNA and RNA were detected in all the recipients except M467 (Figures 5C–F).

## Discussion

A primary barrier to an HIV “cure” rests in the establishment of a stably integrated nonproductive infection that is termed viral latency. Elucidation of location and extent of this viral reservoir is imperative if viral elimination is to be achieved. Current methods to quantitate latent HIV-1 infection relies principally on the use of nucleic acid polymerase chain reaction (PCR) and qVOA-based assays. While both are sensitive and specific both fall short in reflecting the absolute viral reservoir as it exists at extremely low levels (Procopio et al., 2015; Bruner et al., 2019). The lack of assay sensitivity was seen from allogeneic hematopoietic stem cell transplant (HSCT) recipients who were found to be HIV-1 negative by conventional PCR and qVOA by tests of peripheral blood and rectal tissue biopsy for time periods of up to years before analytic treatment interruption (ATI). Unfortunately, both patients experienced viral resurgence (Henrich et al., 2014). In the case of the “Mississippi baby,” ART was initiated 30 h after birth. While the baby remained virus free as examined by PCR and qVOA tests, HIV-1 rebounded after 27 months following ART interruption (Luzuriaga et al., 2015). From these studies it is argued that, ATI is the ultimate standard to justify HIV-1 cure. Both above cases emphasize that more sensitive assays are urgently required at the age of pursuing HIV-1 eradication. However, to date, studies that involved thousands of individuals undergoing treatment interruption, only two cases were considered free of HIV-1 infection while others unexclusively experienced viral rebound (Hutter et al., 2009; Dubrocq and Rakhmanina, 2018; Gupta et al., 2019). Treatment interruption can be avoided if more sensitive assays for viral latency are made available.

An ideal model should have a shortened disease course for completion of therapeutic testing in a limited time and should possess immune cell markers that are readily translatable to humans. That encouraged the creation of immunodeficient rodents that accommodate human xenografts permitting study of diseases exclusively in human such as HIV-1. Mouse models have extended our understanding and prepared us with the therapy for this once life-threatening disease. From past 5 years, researchers employed these models to develop an *in vivo* VOA by engrafting blood samples from HIV-1 infected patient into either non-humanized or humanized mice (Metcalf Pate et al., 2015; Charlins et al., 2017; Henrich et al., 2017; Yuan et al., 2017; Salgado et al., 2018; Schmitt and Akkina, 2018). HIV-1 was successfully recovered in mouse models engrafted from HIV-1 infected and ART suppressed donors. More importantly, mVOA was able to capture latent reservoirs that were not detected by qVOA (Metcalf Pate et al., 2015; Charlins et al., 2017), revealing its higher sensitivity which could facilitate the assessment of viral elimination.

As a valid animal model, humanized mice have been tested with complex and advanced HIV-1 cure strategies, which require clearance of proviral DNA. Recently, we developed a combinational strategy for HIV-1 elimination (Dash et al., 2019). To confirm the clearance of viral infection, splenocytes and bone marrow from these animals were adoptive transferred into naïve humanized mice. HIV-1 was recovered for one of the donors (M3324) but not in M3319 and M3336. Tissue HIV-1 analysis demonstrated absence of virus in M3319 and M3336 but low levels of viral replication in M3324. This demonstrated that replication-competent virus persists even when peripheral VL is undetected. Over 2 months of HIV-1 remission after ATI rendered M3324 as a post-treatment controller (PTC) in which long-term viral control was achieved upon treatment interruption especially in individuals who initiated ART early (Saez-Cirion et al., 2013). However, replication-competent HIV-1 persisted in PTCs that were detected and led to viral rebound (Henrich et al., 2014; Luzuriaga et al., 2015). These residual viruses can be captured by mVOA. To reproduce clinical reports where human donors remained on suppressive treatment when adoptive transfer was performed, a second study was conducted where mouse donors were during ART treatment demonstrating VL at or below the detection limit. In these experiments HIV-1 was recovered from both spleen and PBMCs (De Scheerder et al., 2019; Hataye et al., 2019). In donor M387 in which plasma VL was below the detection limit, HIV-1 was recovered from PBMCs but not from splenocytes. This could mainly be attributed to low engraftment (Charlins et al., 2017). Interestingly, we found that engrafted PBMCs from M387 were less than in splenocytes (0.3 vs. 1.5 million), implying that the HIV-1 presence was evenly distributed among compartments and latency reversal is stochastic (Razooky et al., 2015; Hansen et al., 2018). A second possibility for the failure of HIV-1 recovery is small viral reservoir size. In the present study, treatment was initiated at 2 weeks after HIV-1 challenge when viral latency was initially established (Su et al., 2019). In contrast, human donors from previous reports showed a large viral reservoir size (Strain et al., 2005; Buzon et al., 2014; Metcalf Pate et al., 2015; Charlins et al., 2017). Limited residual HIV-1 reservoirs also explained the absence of detectable tissue HIV-1 in M387. Similar observations were seen when a person started ART within 10 days of infection and remained HIV-1 undetected. Here a total of 530 million CD4+ T cells were infused into 10 mice and only a single animal showed low-level viremia of 201 copies/mL (Henrich et al., 2017). This demonstrated the sensitivity limitation of the mVOA assay. We posit that the assay sensitivity could be improved by incorporating cell stimulation before transfer or by CD8+ T cells depletion after engraftment.

A limitation of the current study is the lack of comparisons made between qVOA and mVOA for assay sensitivity. In conclusion, we described a humanized mouse-to-mouse mVOA which successfully recapitulated what was reported when human samples were employed for adoptive transfer. HIV-1 was captured by recipients from ART suppressed donors when much fewer human cells were engrafted. We were able to interrogate individual tissue HIV-1 reservoirs. This can be extended to any tissue or cell type. Successful recovery of tissue HIV-1 reservoirs from virally suppressed donors emphasizes the importance of latent infections as an obstacle to cure (Rose et al., 2018). Future work need to be conducted to further refine mVOA in order to provide further validations for the elimination of HIV-1 infection.

## Data Availability Statement

All datasets generated for this study are included in the article/Supplementary Material.

## Ethics Statement

The animal study was reviewed and approved by University of Nebraska Medical Center Institutional Animal Care and Use Committee.

## Author Contributions

PD and HG conceived the idea. PD designed the experiments. PD and HS performed the virologic, immunologic, molecular biology experiments, interpreted and plotted the datasets, and prepared the figures. SS processed the animal tissues and performed immunohistochemical testing. SG and LP provided the humanized mice used in the study. KK and RK designed the CRISPR-Cas9 constructs used in the study and analyzed the animal tissues. The manuscript was written by PD, HS, and HG. Manuscript editing was performed by HG, SG, and LP. Funding grant support and project oversight was provided by HG.

### Conflict of Interest

The authors declare that the research was conducted in the absence of any commercial or financial relationships that could be construed as a potential conflict of interest.
