# Supplementary material for: Amplification of Replication Competent HIV-1 by Adoptive Transfer of Human Cells From Infected Humanized Mice
Source: Front Cell Infect Microbiol. 2020 Feb 11;10:38. doi: 10.3389/fcimb.2020.00038 (PMC7026001; doi:10.3389/fcimb.2020.00038)
Supplement: Supplementary file 1 [file Data_Sheet_1.pdf]

Amplification of Replication Competent HIV-1 by Adoptive Transfer of Human Cells from Infected Humanized mice

SUPPLEMENTARY MATERIAL

Hang Su[1], Sruthi Sravanam[1], Santhi Gorantla[1], Rafal Kaminski[2], Kamel Khalili[2], Larisa Poluektova[1], Howard E. Gendelman[1, 3, *‡] and Prasanta K. Dash[1, *]

[1] *Department of Pharmacology and Experimental Neuroscience, College of Medicine, University of Nebraska Medical Center, Omaha, NE, USA*

[2] *Department of Neuroscience, Center for Neurovirology, Lewis Katz School of Medicine at Temple University, Philadelphia, PA, USA*

[3] *Department of Pharmaceutical Sciences, College of Pharmacy, University of Nebraska Medical Center, Omaha, NE, USA*

Running Title: Humanized mouse viral outgrowth Assay

***Corresponding authors:** Prasanta K. Dash, Ph.D., email: pdash@unmc.edu; phone: 402-559-8925; and Howard E. Gendelman, M.D., email: hegendel@unmc.edu; phone: 402-559-8920

‡**Communicating author** (for submission and review): Howard E. Gendelman, M.D., Department of Pharmacology and Experimental Neuroscience, University of Nebraska Medical Center, Omaha, NE, USA; phone: 402-559-8920; fax: 402-559-3744; email: hegendel@unmc.edu

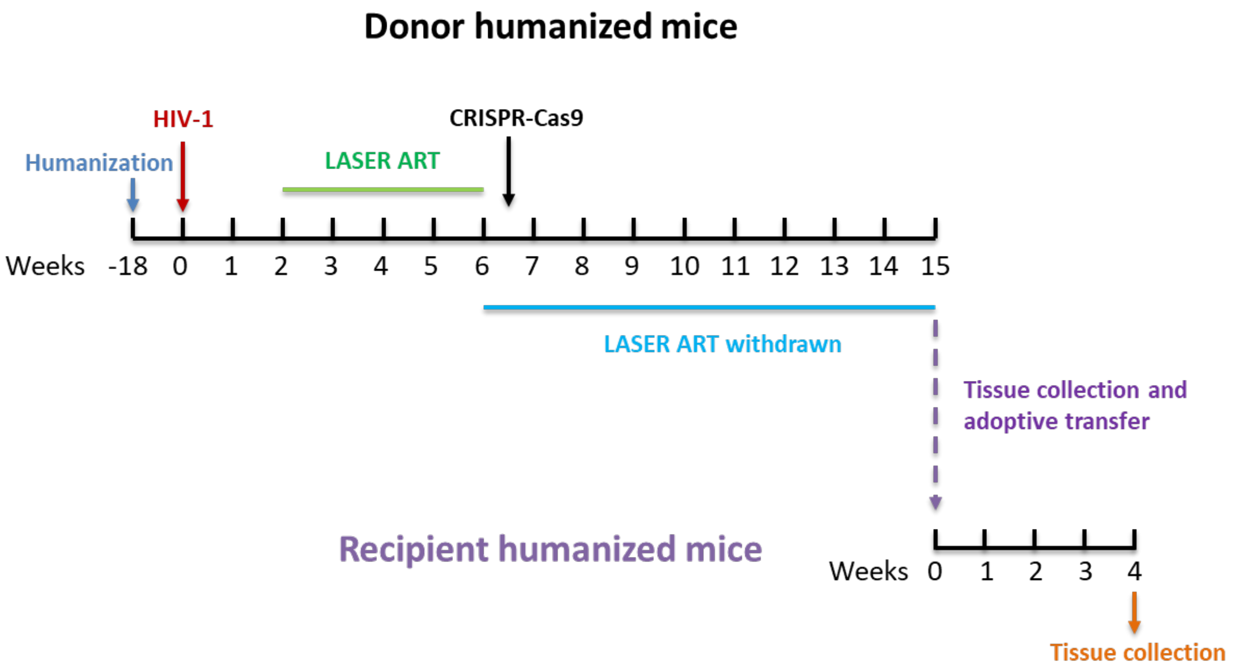

Figure S1. The experimental scheme for infected donor humanized mice that received LASER ART and CRISPR-Cas9 followed by adoptive transfer. In brief, donor humanized mice were first infected with HIV-1$_{NL4\text{-}3}$ or HIV-1$_{ADA}$ for 2 weeks. Animals then received LASER ART which contained a combination of nanoformulated long-acting DTG, RPV, ABC, and 3TC followed with single administration of CRISPR-Cas9. After 8 weeks of CRISPR-Cas9 which equaled to 9 weeks of LASER ART discontinuance, donor humanized mouse splenocytes and BM cells were isolated and separately engrafted to respective naïve humanized mice. The recipient animals were maintained for 4 weeks to monitor HIV-1 recovery.

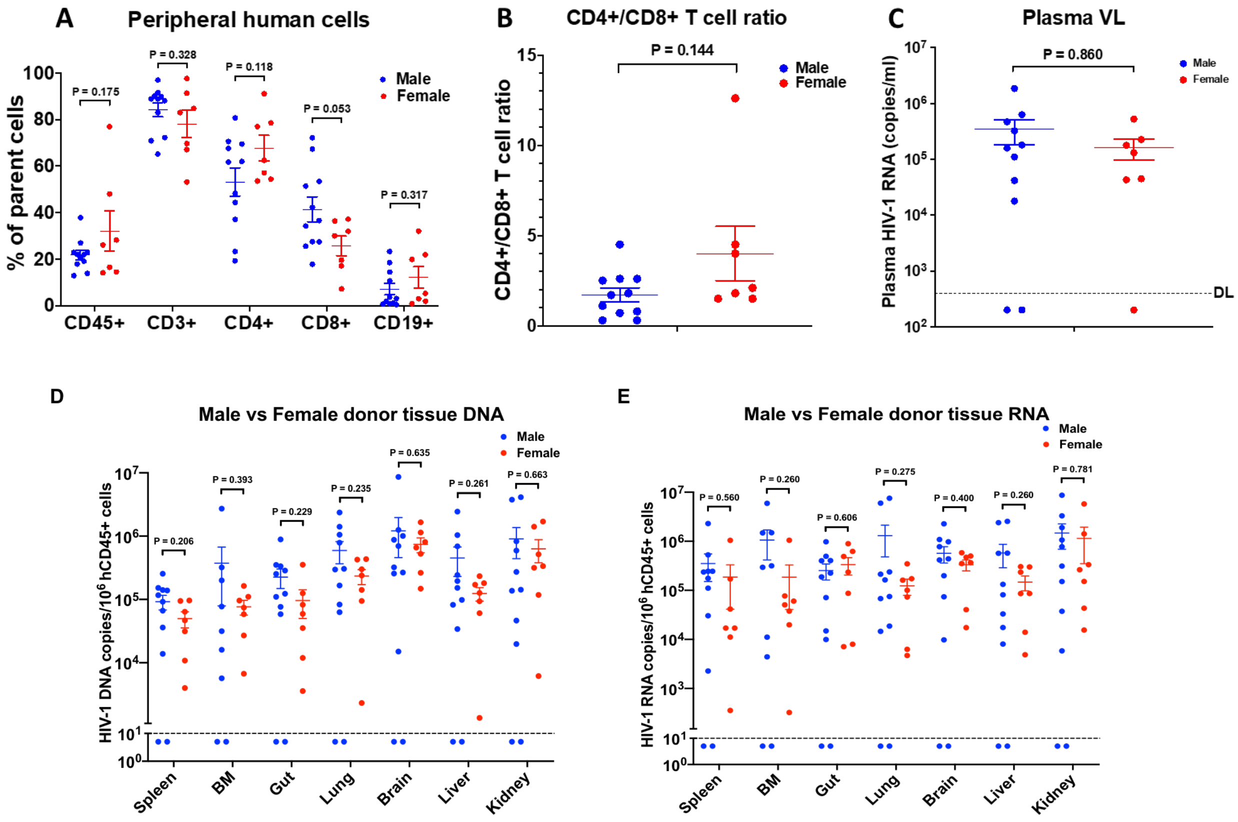

Figure S2. Descriptions of donor humanized mice. Detailed immune and viral profile of individual donor NSG-humanized mice (n = 18) was displayed as scatter dots according to sex. Male (n = 11, blue) and female (n = 7, red) donors were compared on (**A**) peripheral human CD45+, CD3+, CD4+, CD8+, and CD19+ cell levels, (**B**) CD4+/CD8+ T cell ratio, (**C**) plasma viral load (pVL), (**D**) tissue HIV-1 DNA and (**E**) tissue HIV-1 RNA distributed across spleen, BM, gut, lung, brain, liver, and kidney. Data are expressed as mean ± SEM. The detection limit (DL) of plasma VL is 400 HIV-1 RNA copies/ml. The detection limit of tissue viral analysis is below 10 HIV-1 copies/10^6 human CD45+ cells as measure using real time semi-nested qPCR analysis. The dotted line indicates the level of detection.

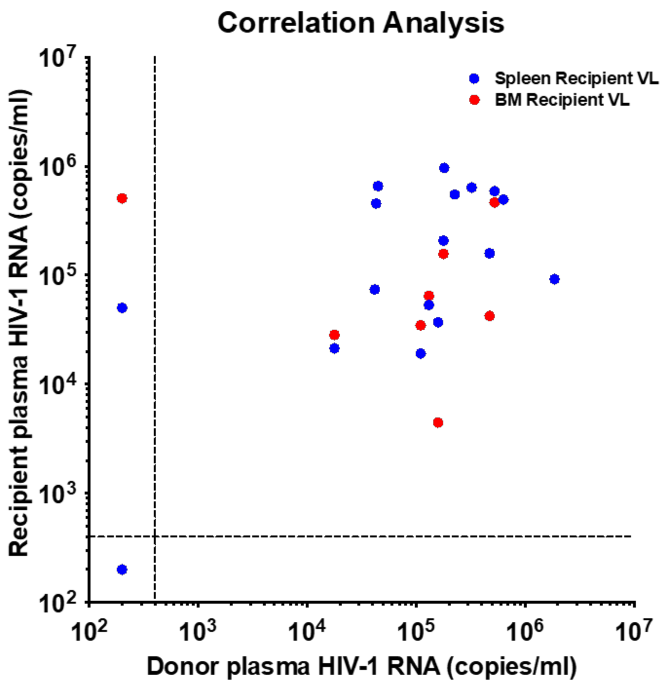

	Spleen Recipient VL (n=18)	BM Recipient VL (n=10)
Donor VL (n=18)	r = 0.607	r = 0.437
	P = 0.008	P = 0.206

Figure S3. Correlations between donor and recipient mouse plasma VL. Spearman's rank correlation tests were conducted to investigate associations between donor humanized mouse plasma VL and respective SR plasma VL (blue) and respective BR plasma VL (red). The Spearman correlation coefficient (r) and p value for each analysis were displayed. The detection limit of plasma VL is 400 HIV-1 RNA copies/ml.

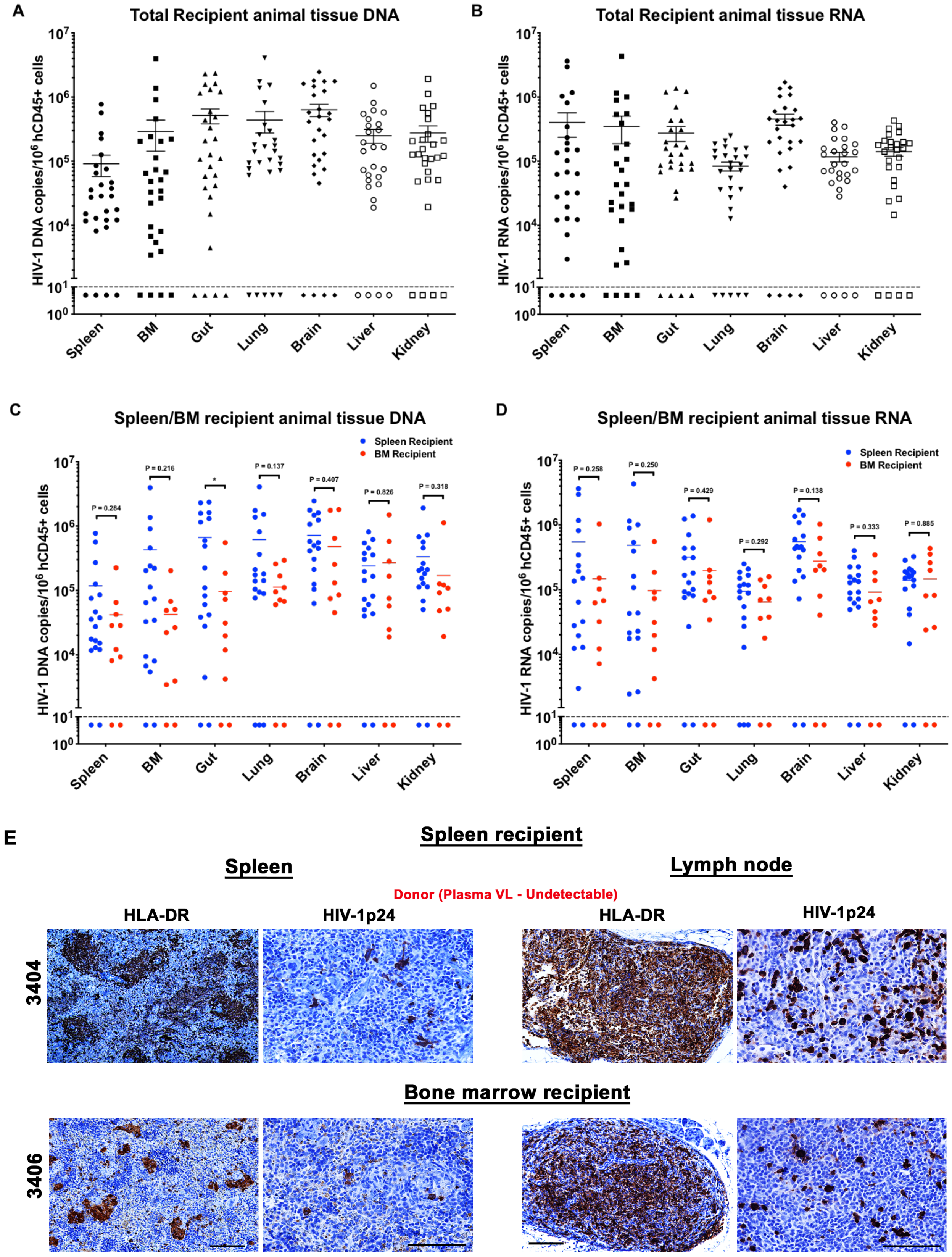
A
Total Recipient animal tissue DNA
HIV-1 DNA copies/10^6 hCD45+ cells
Spleen
BM
Gut
Lung
Brain
Liver
Kidney
B
Total Recipient animal tissue RNA
HIV-1 RNA copies/10^6 hCD45+ cells
C
Spleen/BM recipient animal tissue DNA
Spleen Recipient
BM Recipient
P = 0.284
P = 0.216
*
P = 0.137
P = 0.407
P = 0.826
P = 0.318
D
Spleen/BM recipient animal tissue RNA
P = 0.258
P = 0.250
P = 0.429
P = 0.292
P = 0.138
P = 0.333
P = 0.885
E
Spleen recipient
Spleen
Lymph node
Donor (Plasma VL - Undetectable)
HLA-DR
HIV-1p24
3404
Bone marrow recipient
3406

Figure S4. HIV-1 DNA and RNA levels and detection of HIV-1p24 in selected tissues from recipient humanized mice. (A) Tissue HIV-1 DNA and (**B**) tissue HIV-1 RNA in total recipient (TR) were established in spleen, BM, gut, lung, brain, liver, and kidney. **(C)** Tissue HIV-1 DNA and (**D**) tissue HIV-1 RNA in subgroups spleen recipient (SR) (blue) and bone marrow recipient (BR) (red) were displayed. The detection limit is below 10 HIV-1 copies/10^6 human CD45+ cells as measured by real-time semi-nested qPCR assays. Data are expressed as mean ± SEM and considered * statistically significant, at $p < 0.05$. (**E**) A representative photomicrographic analysis of spleen and lymph node samples from spleen and BM recipient of M3324 was presented. Tissues were collected at necropsy then fixed with 4% PFA and paraffin embedded for next step. Five μM thick sections were cut and then stained with antibodies specific for human HLA-DR and HIV-1p24. Representative images from each group were selected and pictures were captured for both (shown as brown dots) from individual animals. The recipient mice numbers are presented on the left panel. Scale bar 10μM and 40 μM respectively for HLA-DR and HIV-1p24 analysis.

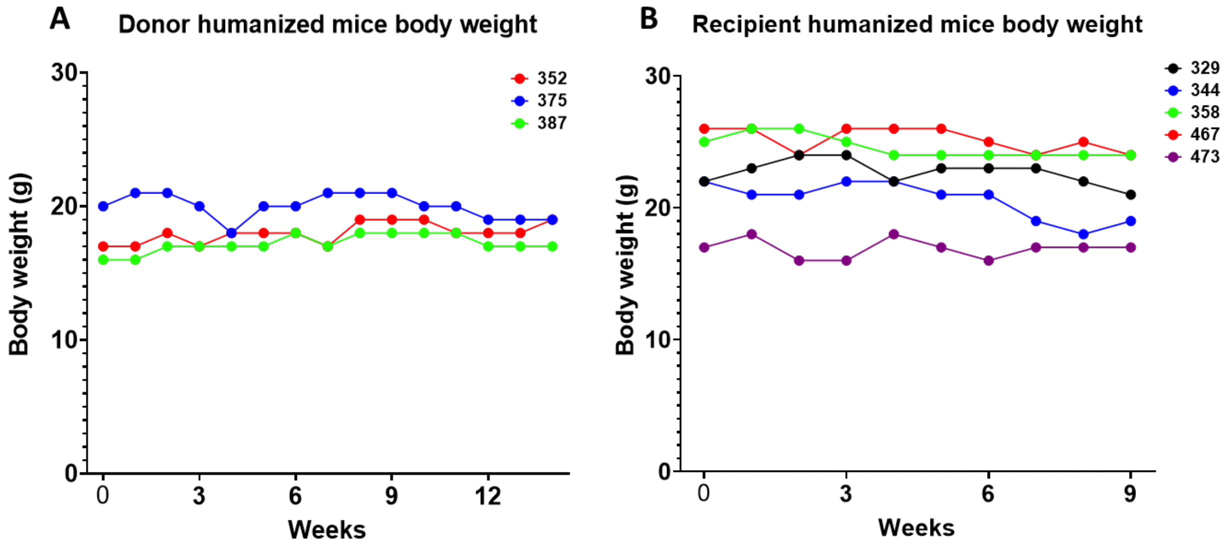

Figure S5. Body weights of donor humanized mice during LASER ART and recipient humanized mice after adoptive transfer. (A) Body weight of 3 individual donor humanized mouse was recorded weekly throughout the study. M352, M375, and M387 were labeled with red, blue, and green line, respectively. **(B)** Body weight of all 5 individual recipient humanized mouse was recorded weekly throughout the study. M344 (blue), M473 (purple), and M467 (red) were splenocytes recipients from M352, M375, and M387, respectively. M358 (green) and M329 (black) were PBMCs recipients from M375 and M387, respectively.
