# Supplementary material for: Amplification of Replication Competent HIV-1 by Adoptive Transfer of Human Cells From Infected Humanized Mice
Source: Front Cell Infect Microbiol. 2020 Feb 11;10:38. doi: 10.3389/fcimb.2020.00038 (PMC7026001; doi:10.3389/fcimb.2020.00038)
Supplement: Supplementary file 2 [file Data_Sheet_2.doc]

**Table S1. Descriptors of donor humanized mice**

| **Animal ID**  **(n=18)** | **Sex** | **Age (weeks)** | **Peripheral hCD45 (%)** | **HIV-1**  **strain** | **Treatment** | **Plasma HIV RNA (copies/ml)** |
| --- | --- | --- | --- | --- | --- | --- |
| 3136 | F | 44 | 28.3 | NL4-3 | LASER ART | 42800 |
| 3181 | M | 42 | 22.9 | NL4-3 | LASER ART | 632000 |
| 3182 | M | 42 | 22.1 | NL4-3 | LASER ART | 324000 |
| 3198 | M | 37 | 17.9 | NL4-3 | Dual | 181000 |
| 3199 | M | 37 | 19.1 | NL4-3 | Dual | 1860000 |
| 3201 | F | 37 | 48.0 | NL4-3 | Dual | 44640 |
| 3319 | M | 36 | 27.0 | ADA | Dual | **ND** |
| 3322 | M | 36 | 20.6 | ADA | Dual | 41600 |
| 3324 | F | 34 | 77.0 | ADA | Dual | **ND** |
| 3327 | M | 39 | 37.8 | ADA | Dual | 17800 |
| 3328 | M | 39 | 12.9 | ADA | LASER ART | 159000 |
| 3333 | M | 34 | 21.9 | ADA | LASER ART | 110000 |
| 3336 | M | 34 | 13.9 | ADA | Dual | **ND** |
| 3353 | M | 34 | 23.0 | ADA | Dual | 472000 |
| 3357 | F | 34 | 16.5 | ADA | None | 226000 |
| 3359 | F | 34 | 14.2 | ADA | None | 131000 |
| 3636 | F | 45 | 14.5 | ADA | Dual | 524000 |
| 3637 | F | 45 | 26.1 | ADA | Dual | 178000 |

Donor hu-HSC mouse number and sex (F, female; M, male) are listed. Animals were virus-infected with HIV-1_NL4-3_ or HIV-1_ADA_, followed by no treatment (none), long acting slow effective release antiretroviral therapy [LASER ART containing prodrugs of dolutegravir (DTG), lamivudine (3TC) and abacavir (ABC) and nanoformulated rilpivirine, RPV], or LASER ART and CRISPR/Cas9 (dual). Animals were sacrificed at 8 weeks after CRISPR/Cas9 injection, which equaled to 9 weeks after the final dose of LASER ART. Hu-HSC mouse age, peripheral human CD45+ (hCD45+) cell numbers, and plasma HIV-1 RNA counts at sacrifice are summarized. ND, not detected <400 copies/ml.

**Table S2. Descriptors of recipient humanized mice**

| **Donor ID (n=18)** | **Spleen recipient ID (n=18)** | **Sex** | **Age (weeks)** | **hCD45 before**  **transfer**  **(%)** | **Engrafted cell count (million)** | **End point plasma HIV RNA (copies/**  **ml)** | **BM ID (n=10)** | **Sex** | **Age (weeks)** | **hCD45 before transfer (%)** | **Engrafted cell count (million)** | **End point plasma HIV RNA (copies/ml)** |
| --- | --- | --- | --- | --- | --- | --- | --- | --- | --- | --- | --- | --- |
| 3136 | 3361 | F | 25 | 62.8 | 16.0 | 455000 |  |  |  |  |  |  |
| 3181 | 3355 | F | 25 | 37.1 | 12.3 | 495000 |  |  |  |  |  |  |
| 3182 | 3360 | F | 25 | 53.5 | 14.7 | 640000 |  |  |  |  |  |  |
| 3198 | 3323 | M | 27 | 53.3 | 12.9 | 966000 |  |  |  |  |  |  |
| 3199 | 3347 | F | 25 | 61.8 | 12.0 | 92200 |  |  |  |  |  |  |
| 3201 | 3362 | F | 25 | 55.7 | 19.8 | 657000 |  |  |  |  |  |  |
| 3319 | 3432 | F | 31 | 34.3 | 6.5 | ND | 3425 | F | 30 | 34.9 | 7.0 | ND |
| 3322 | 3433 | F | 31 | 51.4 | 9.6 | 74200 |  |  |  |  |  |  |
| 3324 | 3404 | M | 29 | 49.7 | 12.7 | 50400 | 3406 | F | 29 | 15.2 | 10.4 | 508000 |
| 3327 | 3428 | F | 33 | 26.4 | 5.1 | 21400 | 3430 | M | 33 | 14.2 | 10.7 | 28400 |
| 3328 | 3354 | F | 37 | 15.2 | 6.1 | 37200 | 3351 | M | 37 | 15.5 | 10.0 | 4460 |
| 3333 | 3422 | F | 30 | 24.3 | 9.0 | 19200 | 3407 | F | 30 | 27.2 | 5.2 | 34800 |
| 3336 | 3434 | F | 31 | 47 | 6.5 | ND | 3388 | F | 34 | 18.6 | 6.0 | ND |
| 3353 | 3435 | F | 31 | 25.4 | 9.0 | 159000 | 3416 | M | 30 | 39.8 | 4.3 | 42400 |
| 3357 | 3409 | F | 31 | 24.1 | 9.5 | 552000 |  |  |  |  |  |  |
| 3359 | 3410 | F | 31 | 9.33 | 9.6 | 53400 | 3411 | F | 31 | 28.6 | 8.3 | 64800 |
| 3636 | 3912 | M | 29 | 30.9 | 8.9 | 590000 | 3913 | M | 29 | 35.4 | 9.8 | 466000 |
| 3637 | 3914 | M | 29 | 28.4 | 7.3 | 208000 | 3905 | F | 29 | 42 | 9.1 | 157000 |

The identifier number of individual recipient hu-HSC mouse and corresponding donor hu-HSC mouse are listed. A total of 18 hu HSC mice received splenocytes transfers and 10 hu HSC mice BM cells. Sex (F, female; M, male), age, and peripheral human CD45+ (hCD45+) cell levels of recipient animals before adoptive transfer are summarized. From 4.3 to 19.8 million cells were transferred. Recipient hu-HSC mice were maintained for 4 weeks before sacrifice and end point plasma HIV-1 RNA counts were determined. ND, not detected <400 copies/ml. Blank entries represent no cells.

**Table S3. Peripheral human cell reconstitution and plasma VL of recipient mice**

|  | | **TR (n = 28)** | | **SR (n = 18)** | | **BR (n = 10)** | |
| --- | --- | --- | --- | --- | --- | --- | --- |
|  |  | Pre | Post | Pre | Post | Pre | Post |
| **Peripheral human cells (%)** | **CD45** | 34.4 ± 2.9 | 34.8 ± 3.1 | 38.4 ± 3.9 | 39.1 ± 3.9 | 27.1 ± 3.4 | 26.9 ± 4.0 |
|  | **CD3** | 58.5 ± 4.7 | 67.5 ± 3.5 | 62.7 ± 5.9 | 69.9 ± 4.2 | 51.0 ± 7.8 | 63.2 ± 6.4 |
|  | **CD4** | 70.4 ± 1.7 | 45.8 ± 4.1 | 71.4 ± 1.7 | 40.0 ± 5.6 | 68.5 ± 3.8 | 56.2 ± 3.7 |
|  | **CD8** | 25.6 ± 1.5 | 47.0 ± 3.7 | 24.9 ± 1.6 | 52.2 ± 5.1 | 26.8 ± 3.4 | 37.6 ± 3.1 |
|  | **CD19** | 33.3 ± 4.6 | 23.1 ± 3.2 | 28.8 ± 5.9 | 20.7 ± 3.9 | 41.2 ± 7.3 | 27.5 ± 5.7 |
| **CD4/CD8** | | 3.2 ± 0.3 | 1.4 ± 0.2 | 3.2 ± 0.3 | 1.3 ± 0.4 | 3.3 ± 0.7 | 1.7 ± 0.2 |
| **Plasma HIV-1 RNA**  **(x10^5^ copies/ml)** | | / | 2.3 ± 0.5 | / | 2.8 ± 0.7 | / | 1.3 ± 0.6 |

TR, total recipient; SR, spleen recipient; BR, bone marrow recipient; Pre, pre-adoptive transfer; Post, post-adoptive transfer. Data are expressed as mean ± SEM.

**Table S4. Donor humanized mice maintained under effective treatment**

| **Animal ID** | **Sex** | **Age (weeks)** | **Peripheral hCD45 (%)** | **Peripheral hCD4 (%)** | **Spleen hCD45 (%)** | **Spleen hCD4 (%)** | **Plasma HIV RNA (copies/ml)** |
| --- | --- | --- | --- | --- | --- | --- | --- |
| 352 | F | 36 | 34.3 | 37.4 | 45.6 | 54.7 | 400 |
| 375 | M | 37 | 32.7 | 69.5 | 16.6 | 65.2 | **ND** |
| 387 | F | 31 | 29.0 | 48.2 | 25.7 | 44.6 | 700 |

Donor hu-HSC mouse number and sex (F, female; M, male) are listed. Animals were virus-infected with HIV-1_ADA_, followed by 3-month of LASER ART consisting of nanoformulated long-acting cabotegravir (CAB), lamuvidine (3TC) and abacavir (ABC) and nanoformulated rilpivirine (RPV). Adoptive transfer of donor splenocytes and PBMCs was then performed to monitor HIV-1 recovery. Hu-HSC mouse age, human CD45+ and human CD4+ T cell proportions in peripheral blood and spleen, plasma HIV-1 RNA counts at sacrifice are summarized. ND, not detected <400 copies/ml.

**Table S5. Recipient humanized mice from HIV-1 suppressed donors**

| **Donor ID** | **Spleen recipient ID** | **Sex** | **Age (weeks)** | **hCD45 engraftment (million)** | **hCD4 engraftment (million)** | **End point plasma HIV RNA (copies/ml)** | **PBMCs recipient ID** | **Sex** | **Age (weeks)** | **hCD45 engraftment (million)** | **hCD4 engraftment (million)** | **End point plasma HIV RNA (copies/ml)** |
| --- | --- | --- | --- | --- | --- | --- | --- | --- | --- | --- | --- | --- |
| 352 | 344 | F | 46 | 1.8 | 1 | 470000 |  |  |  |  |  |  |
| 375 | 473 | F | 25 | 1.3 | 0.8 | 47200 | 358 | M | 35 | 0.6 | 0.4 | 1530000 |
| 387 | 467 | M | 25 | 1.5 | 0.7 | **ND** | 329 | M | 46 | 0.3 | 0.2 | 664000 |

The identifier number of individual recipient hu-HSC mouse and corresponding donor hu-HSC mouse are listed. Of 5 recipient humanized mice in total, 3 received splenocytes transfers and 2 received PBMCs engraftment. Sex (F, female; M, male), age, the engrafted human CD45+ (hCD45+) cell counts and equivalent human CD4+ (hCD4+) T cell counts are summarized. Recipient hu-HSC mice were maintained for 9 weeks before sacrifice and end point plasma HIV-1 RNA counts were determined. ND, not detected <400 copies/ml. Blank entries represent no cells.
